# Supplementary material for: T4-like Bacteriophages Isolated from Pig Stools Infect Yersinia pseudotuberculosis and Yersinia pestis Using LPS and OmpF as Receptors
Source: Viruses. 2021 Feb 13;13(2):296. doi: 10.3390/v13020296 (PMC7917993; doi:10.3390/v13020296)
Supplement: Supplementary file 1 [file viruses-13-00296-s001.pdf]

pharmaceutics

Supplementary Material: Understanding the Salt-Dependent Outcome of Glycine Polymorphic Nucleation

Guangjun Han, Pui Shan Chow and Reginald B.H. Tan

Supplementary Materials—Properties and PXRDs of α-, β- and γ-glycine polymorphs

1. Properties of α-, β- and γ-glycine polymorphs

Some of the properties of the three glycine polymorphs are summarized in Table S1.

Table S1. Properties of α-, β- and γ-glycine crystals.

Property	α-Glycine	β-Glycine	γ-Glycine
space group [1]	$P2_1/n$	$P2_1$	$P3_1$ or $P3_2$
Z (formula units per cell) [1]	4	2	3
unit cell volume (Å^3) [1]	309.6	157.3	235.1
solubility in pure water at 298.15 K (g/(100g water)) [2]	25.03	NA	23.49
solution enthalpy $\Delta_{sol}H_m$ (J mol^{-1}) [3]	14,523 ± 76	14,198 ± 73	14,791 ± 84
sublimation enthalpy ΔH_{sub} at 298.15 K (J mol^{-1} K^{-1}) [4]	140.58	138.09	141.70
isobaric heat capacity C_p at 298.15 K (J mol^{-1} K^{-1}) [4]	89.85	90.46	89.78

2. PXRD patterns and analyses of glycine polymorphs

The simulated PXRD patterns of α-, β- and γ-glycine references are shown in Figure S1-a. Note that Materials Studio's CSD codes of Gly29 (α-form), Gly33 (γ-form) and Gly31 (β-form) were used in PXRD simulation. As an illustration of solid phase analyses, the PXRD patterns of α-glycine crystal samples before and after the test of α-glycine solubility in a glycine aqueous solution in the presence of 1m Na_2SO_4 are presented in Figures S1-b. Similarly, the PXRD patterns of γ-glycine crystal samples before and after the test of γ-glycine solubility in a glycine aqueous solution in the presence of 1m Na_2SO_4 are presented in Figures S1-c.

Comparing the PXRD patterns (Figure S1-b) of α-glycine crystal samples with those (Figure S1-a) of α-, β- and γ-glycine references reveals that the metastable α-glycine crystals remained unchanged in their polymorph before and after α-glycine solubility test through an isothermal method. In a similarly way, comparing the PXRD patterns (Figures S1-a and S1-c) enables us to conclude that the thermodynamically stable γ-glycine crystals (Figure S1-c) also remained unchanged in their polymorph before and after γ-glycine solubility test, which is well expected.

Citation: Han, G.; Chow, P.S.; Tan, R.B.H. Understanding the Salt-Dependent Outcome of Glycine Polymorphic Nucleation. *Pharmaceutics* **2021**, *13*

Publisher's Note: MDPI stays neutral with regard to jurisdictional claims in published maps and institutional affiliations.

Copyright: © 2021 by the authors. Submitted for possible open access publication under the terms and conditions of the Creative Commons Attribution (CC BY) license (http://creativecommons.org/licenses/by/4.0/).

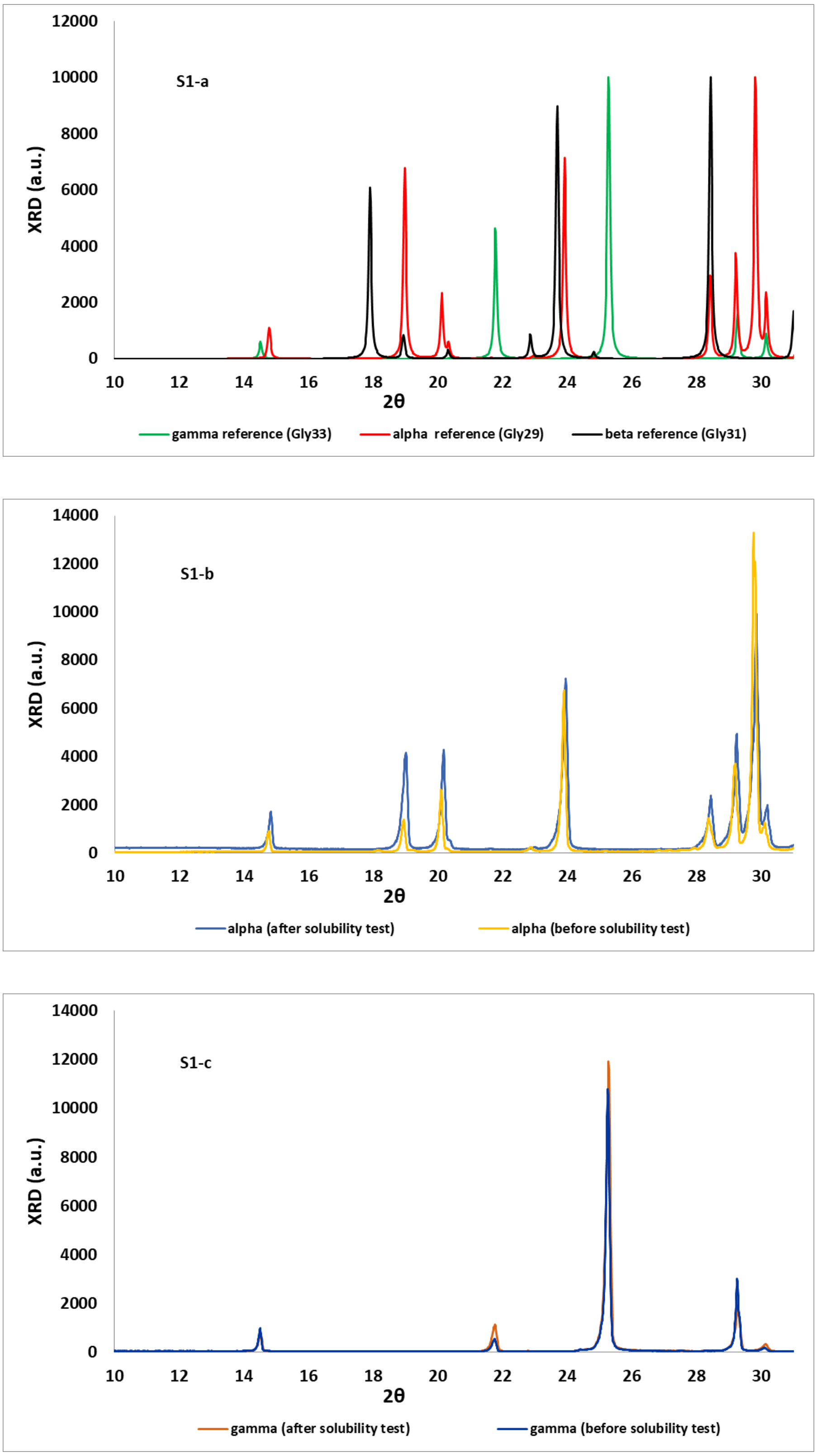

Figure S1. PXRD patterns of α-, β- and γ-glycine references (**S1-a**), α-glycine crystal samples (**S1-b**) before and after the α-glycine solubility test, and γ-glycine crystal samples (**S1-c**) before and after the γ-glycine solubility test.

References

1. Iitaka, Y. The crystal structure of β-glycine. *Acta Cryst*. **1960**, *13*, 35–45.
2. Han, G.; Chow, P.S.; Tan, R.B.H. Understanding the Salt-Dependent Outcome of Glycine Polymorphic Nucleation. Pharmaceutics **2021**, *13*, 262.
3. Xavier, N.F.; Silva, A.M.; Bauerfeldt, G.F. What Rules the Relative Stability of α-, β-, and γ-Glycine Polymorphs? *Cryst. Growth Des.* **2020**, *20*, 4695−4706.
4. Perlovich, G.L.; Hansen, L.K.; Bauer-Brandl, A. The polymorphism of glycine: Thermochemical and structural aspects. *J. Therm. Anal. Calorim.* **2001**, *66*, 699−715.
